# Targeting of the PI3 K/AKT/GSK3β Pathway in Parkinson's Disease: A Therapeutic Blueprint

**DOI:** 10.1007/s12035-025-05113-y

**Published:** 2025-06-05

**Authors:** Raed AlRuwaili, Hayder M. Al-kuraishy, Ali I. Al-Gareeb, Ali K. Albuhadily, Athanasios Alexiou, Marios Papadakis, Mohammed E. Abo-El Fetoh, Gaber El-Saber Batiha

**Affiliations:** 1 Department of Internal Medicine, College of Medicine, Northern Border University, Arar, Saudi Arabia; 2 Department of Clinical Pharmacology and Medicine, College of Medicine, Mustansiriyah University, Baghdad, Iraq; 3 Jabir Ibn Hayyan Medical University, Najaf, Iraq; 4 Department of Research & Development, Funogen, Attiki, Athens 11741 Greece; 5 University Centre for Research & Development, Chandigarh University, Chandigarh-Ludhiana Highway, Mohali, Punjab India; 6 University Hospital Witten-Herdecke, University of Witten-Herdecke, Heusnerstrasse 40, Wuppertal, 42283 Germany; 7 Department of Pharmacology and Toxicology, Faculty of Pharmacy, Egyptian Russian University, Badr City, Cairo 11829 Egypt; 8 Department of Pharmacology and Therapeutics, Faculty of Veterinary Medicine, Damanhour University, Damanhour, AlBeheira 22511 Egypt

**Keywords:** Parkinson's disease, Phosphatidylinositol 3-kinase, Glycogen synthase kinase 3 beta, Brain insulin resistance, Neuroinflammation, Neuroprotection, Metformin, Lithium

## Abstract

Parkinson's disease (PD) is a neurodegenerative disease characterized by progressive motor and non-motor symptoms. PD neuropathology is due to the progressive deposition of mutant alpha-synuclein (α-Syn) in the dopaminergic neurons of the substantia nigra pars compacta (SNpc). This effect initiates oxidative stress, mitochondrial dysfunction, inflammation, and apoptosis of the dopaminergic neurons in the SNpc. PD neuropathology, which is closely associated with inflammatory and oxidative disorders, disrupts different vital cellular pathways. Notably, the current anti-PD medications only relieve the symptoms of PD without averting the underlying neuropathology. Thus, it is advisable to search for novel drugs that attenuate the progression of PD neuropathology. It has been shown that phosphatidylinositol 3-kinase (PI3K), AKT, and glycogen synthase kinase 3 beta (GSK3β) signaling pathways are affected in PD. PI3K/AKT pathway is neuroprotective against the development and progression of PD. However, the over-activated GSK3β signaling pathway has a detrimental effect on PD neuropathology by inducing inflammation and oxidative stress. Dysregulation of the PI3K/AKT/GSK3β signaling pathway provokes brain insulin resistance (BIR), neuroinflammation, and neuronal apoptosis, the hallmarks of PD and other neurodegenerative diseases. However, the mechanistic role of the PI3K/AKT/GSK3β signaling pathway is not fully clarified. Therefore, in this review, we intend to discuss the role of the PI3K/AKT/GSK3β signaling pathway in PD pathogenesis and how PI3K/AKT activators and GSK3β inhibitors have helped effectively manage PD.

## Introduction

**Fig. 1:**
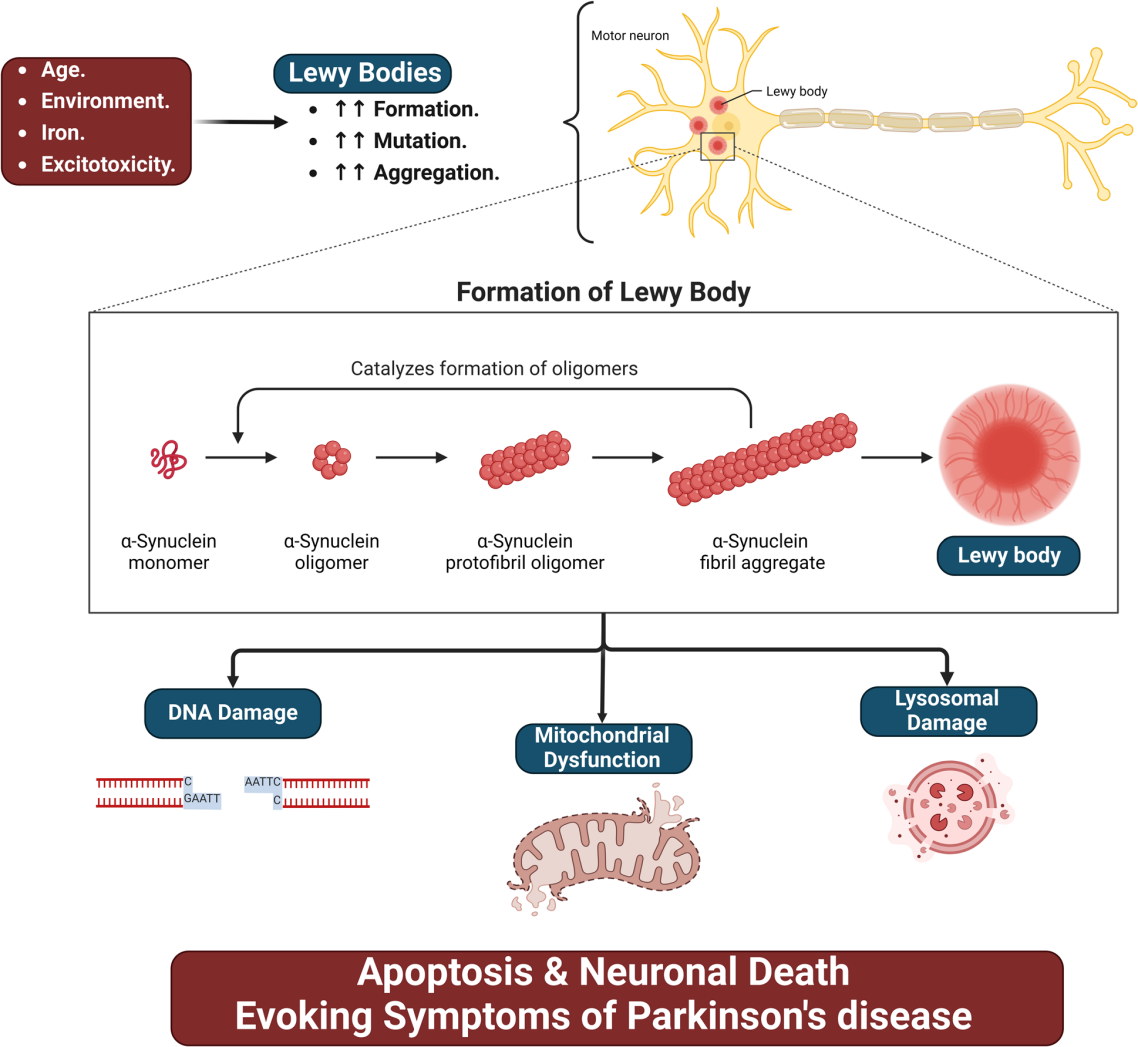
PD neuropathology

Parkinson's disease (PD) is a progressive neurodegenerative disease characterized by motor symptoms such as rigidity, resting tremor, and hypokinesia. PD is also characterized by non-motor symptoms such as cognitive impairment, sleep disorders, neuropsychiatric disorders, and autonomic dysfunctions [1–4]. PD is most common in older age subjects > 65 years and affects < 1% of the population aged 45–54 years. This percentage is augmented to 4% in men and 2% in women aged > 85 years [5]. PD prevalence is thought to be doubling in the following decades to reach a large number [6]. The fundamental causes of PD are principally mysterious; nevertheless, environmental factors such as air pollution and vitamin D deficiency increase PD disease risk in genetically vulnerable persons [7]. Notably, there are two types of PD: sporadic PD, the most common type that forms 90% of PD cases, and familial PD, which represents 5–10% of PD cases. PD neuropathology is due to the progressive deposition of mutant alpha-synuclein (α-Syn) in the dopaminergic neurons of the substantia nigra pars compacta (SNpc). This effect triggers the development of oxidative stress, mitochondrial dysfunction, inflammation, and apoptosis of the dopaminergic neurons in the SNpc [3, 8–17]. In addition, nuclear factor erythroid 2-related factor 2 (Nrf2), which regulates the expression of the NLRP3 inflammasome, improves mitochondrial function and reduces neuroinflammation and neurodegeneration in PD. Furthermore, mitochondrial dysfunction is linked to the aberrant NLRP3 activation in PD. These changes trigger the development of neuroinflammation in PD, and targeting Nrf2 and related signaling pathways can mitigate PD's pathogenesis [18–23]. Consequently, PD neuropathology is multifaceted and linked to varied cellular and subcellular disorders [24, 25] (Fig. 1).

In response to the many inflammatory oxidative stress changes that develop in PD, numerous signaling pathways are triggered, such as phosphatidylinositol 3-kinase (PI3 K), AKT, and glycogen synthase kinase 3 beta (GSK3β) [18]. Recent studies have addressed the PI3 K/AKT/GSK3β signaling pathway in neurodegenerative diseases. The role of PI3 K/AKT/GSK3β signaling pathway in PD is controversial. Commonly expressed PI3 K/AKT/GSK3β signaling pathway regulates neuronal functions and survival. However, the mutant PI3 K/AKT/GSK3β signaling pathway is involved in the induction of progressive neurodegeneration in different neurodegenerative diseases, including PD [3, 26]. PI3 K/AKT is exceedingly altered in PD due to oxidative stress and neuroinflammation and is connected to developing brain insulin resistance (BIR) [27]. PI3 K/AKT improves neuronal survival and metabolism through modulation of GSK3β and forkhead box protein O (FOXO) [28]. Thus, PI3 K/AKT has a neuroprotective effect against the development of neurodegenerative diseases, including PD [29]. Nonetheless, the expression and the activity of GSK3β are increased in PD and contribute to the development of cognitive impairment by exacerbating Aβ and tau protein phosphorylation [30]. Additionally, GSK3β promotes α-Syn aggregation and the death of dopaminergic neurons, characteristic hallmarks of PD pathogenesis [31]. Moreover, GSK3β activation induces neuroinflammatory responses via cytokine production and microglial activation and worsens PD symptoms [30]. Consequently, activation of the PI3 K/AKT signaling pathway and suppression of the GSK3β may be beneficial in managing PD. As Imam Hussain said 1430 years ago,"Knowledge is the vaccine of knowledge, and long experiences are an increase in the mind."Hence, this review aims to discuss the probable role of PI3 K/AKT/GSK3β in PD. In addition, it explores the possible therapeutic efficacy of PI3 K/AKT activators and GSK3β inhibitors in managing PD.

## PI3 K/AKT/GSK3β Signaling Pathway

**Fig. 2:**
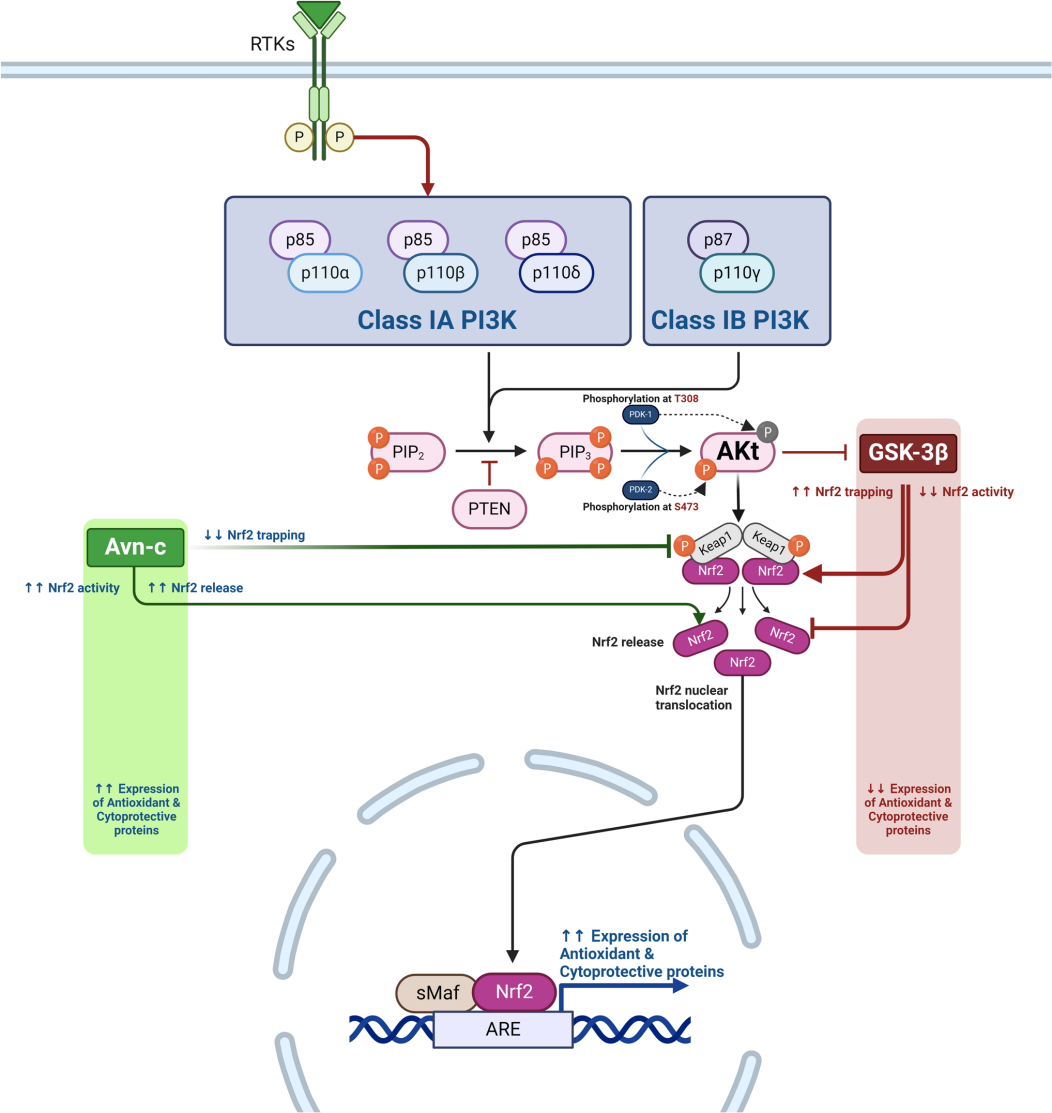
PI3 K/AKT/GSK3β signaling pathway

**Fig. 3:**
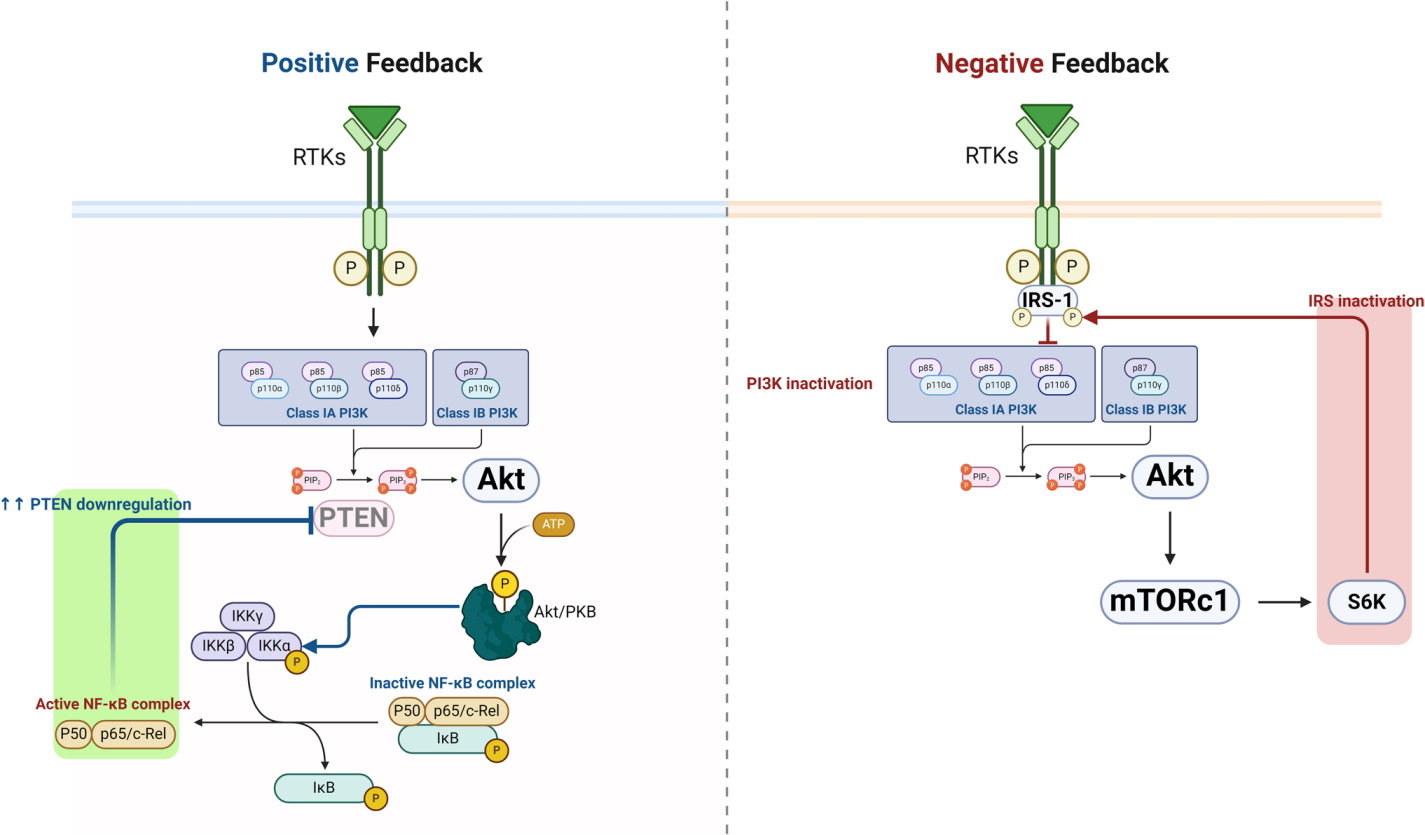
Positive and negative feedback loops in the regulation of PI3 K

PI3 Ks are a family of enzymes that control cell proliferation, growth, differentiation, survival, motility, and intracellular trafficking [32, 33]. PI3 K/AKT is a fundamental pathway for regulating cell proliferation, survival, migration, and metabolism in various physiological and pathological processes. There are four PI3 Ks: Class I PI3 K isoforms (PI3 Kα,β,γ,δ), three Class II PI3 K isoforms (PI3 KC2α, C2β, C2γ), and a single Class III PI3 K (Table 1). The four Class I isoforms synthesize the phospholipid PIP3 [34]. These four isoforms have overlapping functions but are adapted to receive efficient stimulation by particular receptor subtypes. PI3 Kγ is highly expressed in leukocytes and plays a critical role in chemokine-mediated recruitment and activation of innate immune cells at sites of inflammation [35]. PI3 Kδ is also highly expressed in leukocytes and plays a key role in antigen receptor and cytokine-mediated B and T cell development, differentiation, and function. Class III PI3 K synthesizes the phospholipid PI3P, which regulates endosome-lysosome trafficking and the induction of autophagy, pathways involved in pathogen killing, antigen processing, and immune cell survival. Much less is known about the function of Class II PI3 Ks, but emerging evidence indicates they can synthesize PI3P and PI34P2 and regulate endocytosis [35]. This signaling pathway has many molecules, including receptor tyrosine kinase (RTK), phosphatidylinositol-4,5-bisphosphate (PIP2), PI3 K, and AKT [34]. PI3 Ks regulate intracellular signaling intricate in cellular metabolism by activating AKT/protein kinase B [35]. The AKT/protein kinase B is a critical regulator of cellular homeostasis, with diminished AKT activity associated with cellular metabolism dysregulation and cell death. At the same time, AKT over-activation has been linked to inappropriate cell growth and proliferation. AKT expression and enzymatic activity regulate stress response, glucose utilization, and protein metabolism [35]. RTKs are cell surface receptors for growth factors, hormones, and cytokines [36]. PI3 K comprises subunits, the catalytic domain P110 and the regulatory domain P85 [37]. The PI3 K regulatory subunits exist in excess over the p110 catalytic subunits and, therefore, are free in the cell. P110-independent p85 is unstable and exists in a monomer–dimer equilibrium. PI3 K is activated mainly through the regulatory subunit or indirectly by activated adaptor molecules that trigger the activation of RTKs. PIP2 and PIP3 play roles in recruiting AKT to the plasma membrane via the PDKI molecule [38–40]. Insulin, via activation of the protein kinase receptor, activates the expression of PI3 K, further triggering the expression of AKT/protein kinase B [41]. During severe hypoglycemia, energy production is blocked, and an increase in AMP/ATP activates the energy sensor and putative insulin-sensitizer AMP-activated protein kinase (AMPK). AMPK promotes energy conservation and survival by shutting down anabolism and activating catabolic pathways [41]. In sequence, activated AKT stimulates nuclear erythroid-related factor 2 (Nrf2), which activates the antioxidant response element (ARE) [42]. Mounting evidence suggests that Nrf2 could play a neuroprotective role in traumatic brain injury (TBI) models by regulating the expression of numerous antioxidant, anti-inflammatory, and neuroprotective proteins [42]. These changes promote antioxidant and cytoprotection. In addition, AKT inhibits the expression of GSK3β and reduces its inhibitory effect on the Nrf2 [43] (Fig. 2). Moreover, AKT, through activation of nuclear factor kappa B (NF-κB), inhibits phosphates and tensin homolog (PTEN), which regulate the PIP3/PIP2 ratio, leading to the positive feedback loop [44]. However, activation of the mammalian target of rapamycin (mTOR) by AKT increases the expression of S6 K, which induces phosphorylation of insulin receptor substrate (IRS) and inhibition of PI3 K by a negative feedback loop [45] (Fig. 3).

**Table 1:** The isoforms of the PI3 K/AKT

Class type	Functions	Ref
Class I PI3 Ks	Synthesize the phospholipid PIP3, which has overlapping functions and plays a critical role in chemokine-mediated recruitment and activation of innate immune cells at sites of inflammation	[34]
Class II PI3 Ks	Synthesize PI3P and PI34P2 and regulate endocytosis	[35]
Class III PI3 K	Regulates endosome-lysosome trafficking and the induction of autophagy, pathways involved in pathogen killing, antigen processing, and immune cell survival	[35]

**Fig. 4:**
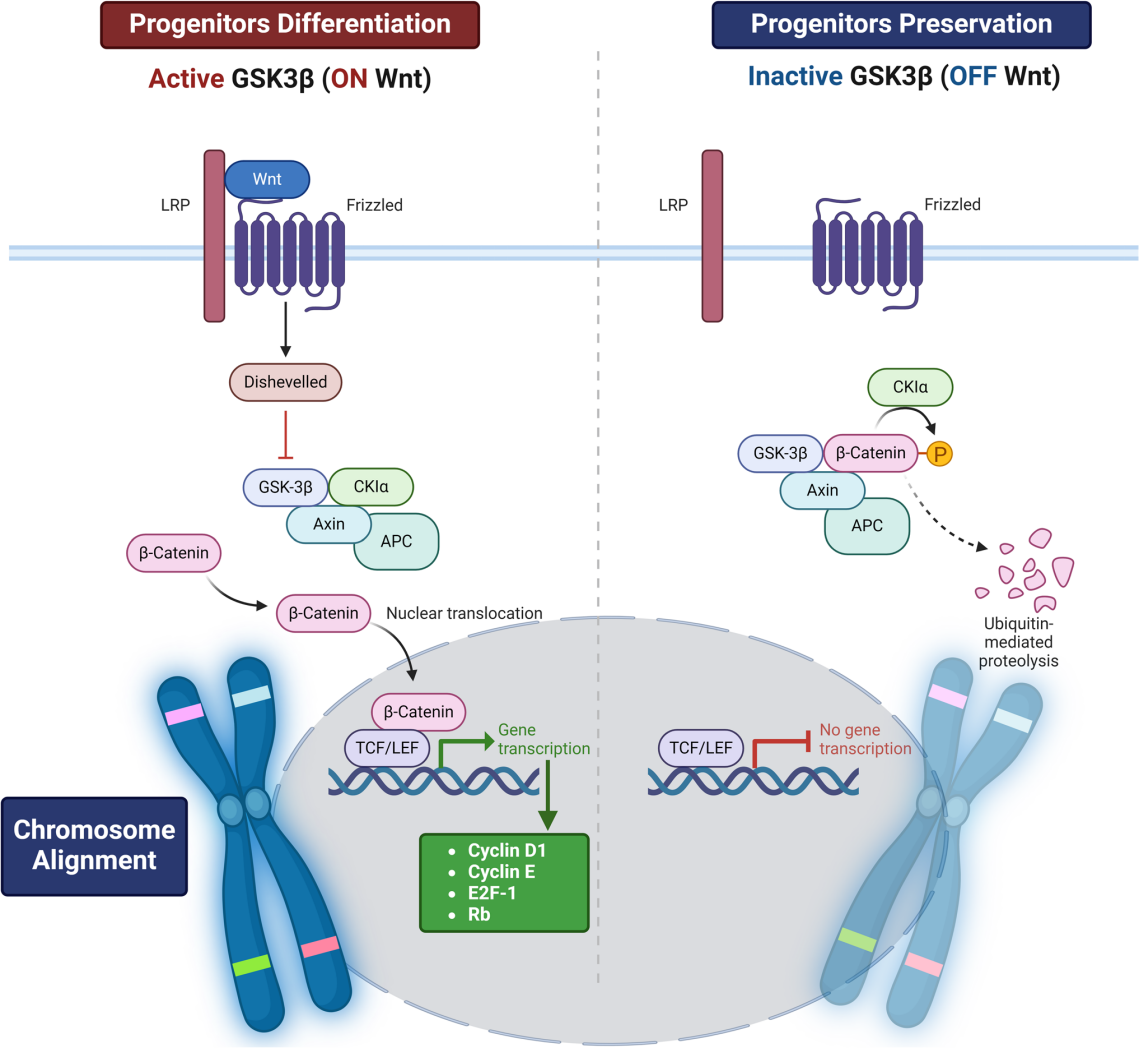
GSK3β signaling pathway

On the other hand, GSK3β is a multifunctional serine/threonine kinase initially identified as a regulator of glycogen metabolism [46]. It plays a key role in regulating numerous signaling pathways, including cellular processes such as cell cycle, inflammation, and cell proliferation. GSK3β regulates cellular metabolic pathways in response to biological stimuli by stimulating glycogen synthase [46]. GSK3β regulates synaptic plasticity, cognitive function, and neurodevelopment [47]. GSK3β plays an essential role in controlling neuronal progenitor proliferation and the establishment of neuronal polarity during development, and the upstream and downstream signals modulate neuronal cytoskeletal reorganization and neuroplasticity[47]. Modulation of GSK3β in brain areas subserving cognitive function has become a significant focus for treating neuropsychiatric and neurodegenerative diseases [47]. As a crucial node that mediates various neuronal processes, GSK3β is proposed to be a therapeutic target for restoring synaptic functioning and cognition, particularly in AD and other neurodegenerative diseases [47]. Unlike GSK3α, which is distributed in the hippocampus, cerebral cortex, and Purkinje cells, GSK3β is distributed in all brain regions [48]. GSK3β regulates oxidative stress and DNA repair via modulation of Nrf2 expression [49]. Pharmacological inhibition or ablation of interneuron GSK3β can restore progenitor cell proliferation, interneuron development, inhibitory/excitatory balance, and hippocampal-dependent behavior [49]. Biochemical targeting of interneuron function may benefit learning deficits caused by oxidative damage [49]. Many signaling intricate in regulating GSK3β, such as Wnt/β-catenin and PI3 K [50]. However, dysregulation and exaggeration of the GSK3β signaling pathway are implicated in the pathogenesis of AD and other neurodegenerative diseases [51]. GSK3β plays a significant role in neuronal apoptosis, and its inhibition decreases the expression of α-Syn, making this kinase an attractive therapeutic target for neurodegenerative disorders [51] (Fig. 4).

## PI3 K/AKT/GSK3β axis in PD

The PI3 K/AKT/GSK3β signaling pathway is dysregulated in PD and intricate in neurotoxicity and neuronal apoptosis [52]. The dysregulated PI3 K/AKT/GSK3β signaling pathway is associated with the development of BIR and neurodegeneration of the dopaminergic neurons in the SNpc and the development of PD dementia [18]. Hence, the overactivation of the mutant PI3 K/AKT/GSK3β signaling pathway is implicated in the pathogenesis of PD. Therefore, targeting this pathway may attenuate neurodegeneration in PD. Consistently, the bioactive component extracted from *Militaris Cordycepin*, known as Cordyceps, inhibits the PI3 K/AKT/mTOR signaling pathway and improves the expression of autophagy proteins in the striatum and SNpc [52]. Activation of neuronal autophagy enhances the clearance of α-Syn, thereby reducing PD progression. Moreover, the PI3 K/AKT signaling pathway inhibits Aβ accumulation and mediates neurotoxicity by suppressing the GSK3β signaling pathway, which induces the formation of amyloid plaque and NFTs [53–55]. Therefore, the PI3 K/AKT signaling pathway has a neuroprotective effect against AD neuropathology by suppressing the Aβ/GSK3β signaling pathway. Furthermore, the PI3 K/AKT signaling pathway controls the expression of mitochondrial membrane permeability proteins Bax and Bcl-2, which are intricate in the induction of apoptosis. In the MPTP PD model, oxidative stress, induction of apoptosis (upregulates the expression of Bax, a proapoptotic protein, and downregulates the expression of anti-apoptotic marker Bcl-2), and loss of dopaminergic neurons, which results in motor impairments, are developed. The antioxidant isookanin regulated Bcl-2/Bax and PI3 K/AKT pathways to reduce mitochondrial damage and cellular apoptosis induced by MPTP in SH-SY5Y cells [56, 57]. Therefore, activating the neuronal PI3 K/AKT pathway regulates neuronal apoptosis and antiapoptotic pathways, subsequently suppressing neurodegeneration in PD.

Of interest, many tyrosine phosphatases, such as GSK3β, which reduce the expression and the activity of the PI3 K/AKT signaling pathway, are up-regulated in PD [30]. GSK-3β-mediated neuroinflammation progression, linking it to regulatory transcription factors and posttranslational modification of NLRP3 inflammasome proteins [30]. GSK3β, a downstream substrate of PI3 K/Akt signaling following induction by insulin and IGF-1, influences AD and PD physiopathology. The genetic overexpression of GSK3β in the cortex and hippocampus results in signs of neurodegeneration and spatial learning deficits in in vivo models [58]. Similarly, protein tyrosine phosphatase 1B (PTP1B), which dephosphorylates IRS-1 and RTKs, reduces the activity of the PI3 K/AKT signaling pathway and is involved in the pathogenesis of PD. PTP1B exaggerates PD neuropathology by inducing oxidative stress, mitochondrial dysfunction, and endoplasmic reticulum (ER) stress by inhibiting the PI3 K/AKT signaling pathway [59]. The PTP1B inhibitor suramin reverses 6-hydroxydopamine (6-OHDA)-induced downregulation of phospho-cAMP response element-binding protein (p-CREB) and brain-derived neurotrophic factor (BDNF) in SH-SY5Y cells. Treatment with suramin could significantly reverse 6-OHDA-induced locomotor deficits and improve tyrosine hydroxylase (TH) via attenuating endoplasmic reticulum (ER) stress biomarkers [59]. These results support that PTP1B could regulate PD via anti-neuroinflammation and antiapoptotic pathways.

Furthermore, the neuronal PTP1B increases α-Syn deposition by activating GSK3β signaling. PTP1B inhibition has been shown to prevent microglial activation, thus exerting a potent anti-inflammatory effect, and has also shown a potential to increase the cognitive process through the stimulation of hippocampal insulin, leptin, and BDNF/TrkB receptors [60]. Therefore, pharmacological inhibition or genetic deletion of PTP1B can reduce the progression of PD through the activation of PI3 K/AKT and inhibition of GSK3β. However, no clinical trials have yet been performed to evaluate the neurological advantages of PTP1B inhibition. Preclinical studies have provided strong evidence that targeting PTP1B could simultaneously allow one to reach different pathophysiological mechanisms. Thus, specific interventions or trials should be designed to modulate PTP1B activity in the brain since it is a promising strategy to decelerate or prevent neurodegeneration in aged subjects with different neurological diseases. Likewise, receptor-type tyrosine-protein phosphatase S (PTPRS) inhibits RTK activity and AKT phosphorylation. The PTPRS is a class of enzymes that catalyze the dephosphorylation of phosphotyrosines in protein molecules. They are involved in cellular signaling by regulating the phosphorylation status of various receptors and signaling molecules within the cell, thereby influencing cellular physiological and pathological processes [61, 62]. The expression of PTPRS is augmented in AD, reducing the activity of the PI3 K/AKT signaling pathway [63].

**Fig. 5:**
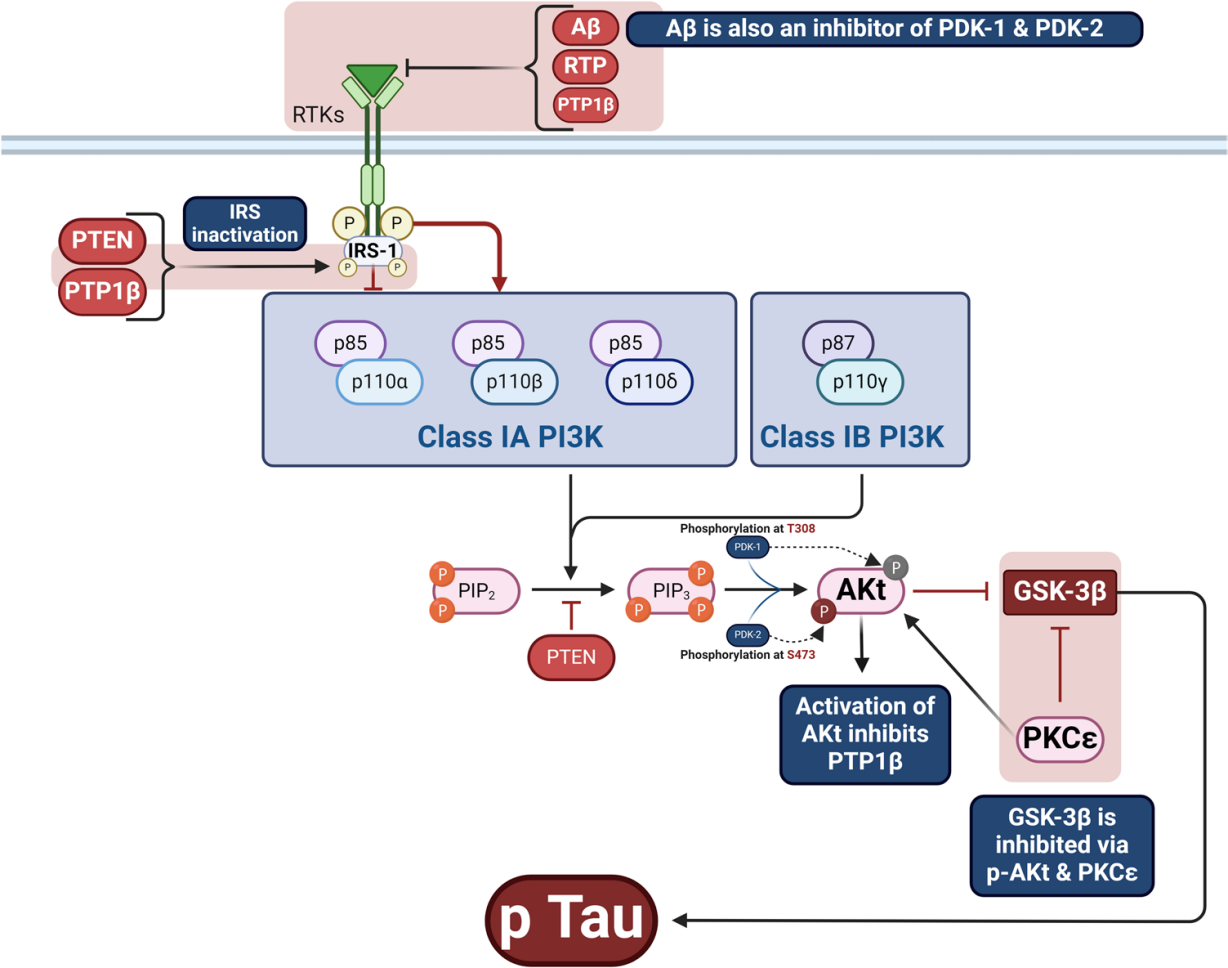
Role of tyrosine phosphatases in PD neuropathology

Additionally, PTEN inhibits the activity of the PI3 K/AKT signaling pathway. Findings from a preclinical study demonstrated that PTEN expression was increased in transgenic PD rat models due to ER stress. PTEN, a negative regulator of brain insulin signaling, inhibits nuclear PI3 K p85, Akt1/2/3, and PIP3 levels in the SNpc region of the PD brain compared to the age-matched controls. A significant decrease in IRβ, IRS1, PI3 K p85, Akt1/2/3, and PIP3 levels and increased GSK3β levels were observed in TH obtained from the SN region of the PD brain compared to the control brain [64]. Thus, alterations in insulin signaling in PD are mediated by the overactivation of neuronal PTEN and the inhibition of the PI3 K/AKT signaling pathway. Consequently, decreasing the nuclear accumulation of PTEN and/or restoring the insulin signaling cascade may halt the neurodegeneration in PD [64]. These observations suggest that the augmentation of tyrosine phosphatases is implicated in the pathogenesis of PD by inhibiting the activity of the PI3 K/AKT signaling pathway (Fig. 5).

Furthermore, downregulation of the PI3 K/AKT signaling pathway is implicated in BIR's pathogenesis, a PD hallmark. It has been illustrated that AKT and phosphorylated AKT are significantly reduced in the SNpc of PD patients. The PI3 K/AKT pathway influences oxidative stress by modulating downstream molecular targets such as GSK-3, mTOR, and FoxO3a. In addition, the PI3 K/AKT/FoxO3a pathway is blocked, resulting in the imbalance of oxidative stress and, eventually, the occurrence of PD. In AD, the PI3 K/AKT signal pathway is involved in forming two special pathological structures, so activating the PI3 K/AKT pathway can delay the progression of AD [64]. Activating the PI3 K/AKT signal pathway can protect neurons against Aβ-induced neurotoxicity. Various targets downstream of this pathway are closely related to the occurrence of the disease. For example, the increase of GSK-3β activity is directly related to the rise of Aβ production and deposition, hyperphosphorylation of tau, and the formation of NFT. During AKT phosphorylation, the phosphorylated protein of GSK-3β is inactivated at the Ser9 site, weakening the Tau protein's hyperphosphorylation and inhibiting the formation of NFT [65]. Therefore, the PI3 K/AKT signaling pathway has a similar neuroprotective effect in PD and AD.

Of interest, α-Syn in PD inhibits the expression and the activity of the PI3 K/AKT signaling pathway, resulting in neuronal apoptosis and progressive neurodegeneration [66]. It has been revealed that α-Syn is a co-regulator of growth factor-induced AKT activation. Elimination of α-Syn reduces the IGF-1-mediated AKT activation. Similarly, mutant α-Syn suppresses the IGF-1-induced AKT activation. Wild-type α-Syn can interact with AKT and enhance the solubility and plasma localization of AKT in response to IGF-1, whereas mutant α-Syn does not interact with AKT. In addition, elevated expression of α-Syn blocks the AKT activation [66]. Furthermore, α-Syn can indirectly inhibit the PI3 K/AKT signaling pathway by activating GSK3β and mTOR in rats [66]. In addition, the deregulation of the PI3 K/AKT signaling pathway by oxidative stress and neuroinflammation exacerbates the pathogenesis of PD [67, 68]. Thus, the PI3 K/AKT signaling pathway is crucial in PD neurodegeneration.

**Fig. 6:**
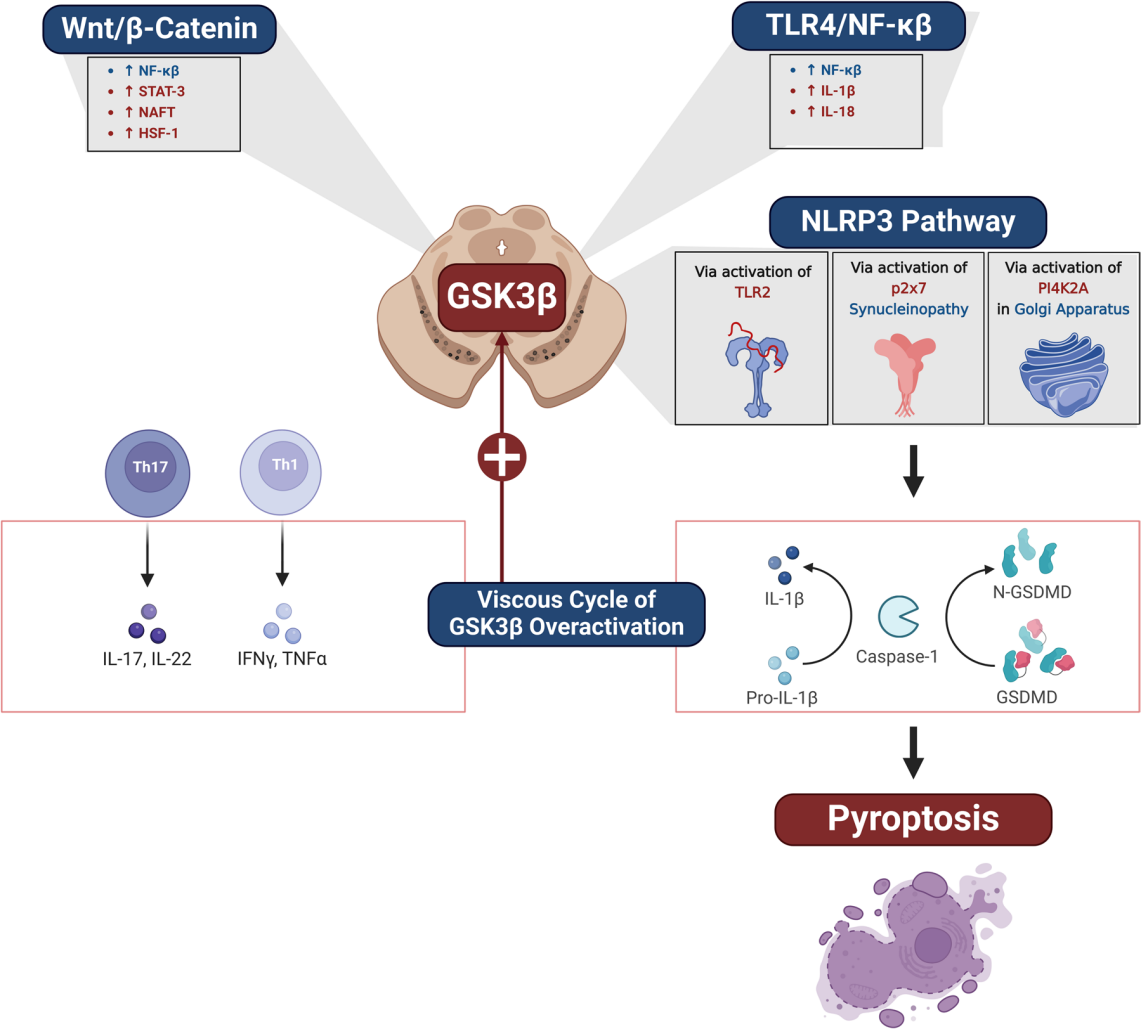
Role of GSK3β in the pathogenesis of PD

Furthermore, the expression of the GSK3β signaling pathway is accelerated and involved in the pathogenesis of PD. Accumulated α-Syn triggers the expression of the *GSK3β* gene with subsequent exaggeration of GSK3β activity, leading to synaptic failure and the development of dementia [58]. In turn, the activated GSK3β promotes the formation and accumulation of α-Syn [69]. The GSK3β is negatively suppressed by insulin and IGF-1, though the development of BIR reduces the inhibitory effect of IGF-1 on the expression of GSK3β [70]. GSK3β and protein phosphatase 2 A mediate tau hyperphosphorylation in AD models. [71]. Thus, activation of GSK3β due to the development of BIR or systemic inflammation exaggerates the severity of PD. It has been reported that the activity of GSK3β is augmented in PD and correlated with oxidative stress and inflammation in the SNpc [72]. In the 6-OHDA PD model, the degeneration of the dopaminergic neurons in the SNpc is mediated by the activated GSK3β [73]. The activated GSK3β in PD attenuates neurogenesis, thereby reducing the recovery following 6-OHDA-induced neurodegeneration of the SNpc [74]. Moreover, activated GSK3β exacerbates PD pathogenesis by inducing the activation of microglia and the release of pro-inflammatory cytokines and, subsequently, the development of neuroinflammation [30, 75]. In addition, the activated GSK3β suppresses neuronal autophagy, attenuating α-Syn clearance in the PD cell model [76]. The activated GSK3β exacerbates PD symptoms by inhibiting dopaminergic neurotransmission and synaptic plasticity in the SNpc [72]. In clinical settings, the expression of the activated GSK3β is augmented in the brains of postmortem PD patients and the peripheral lymphocytes in PD patients compared to healthy controls [77, 78]. Also, the phosphorylated GSK3β in the brains of PD is increased compared to healthy controls, suggesting that the functional activity of GSK3β is augmented in PD [79]. Thus, the role of GSK3β in the pathogenesis of PD is complex and mediated by different signaling pathways (Fig. 6). These findings highlighted that the dysregulated PI3 K/AKT/GSK3β signaling pathway is a crucial duet in the pathogenesis of PD.

## PI3 K/AKT Activators in PD

Activation of the neuroprotective PI3k/Akt with subsequent inhibition of oxidative stress, neuroinflammation, and apoptosis can reduce the development and progression of PD. Different small molecules and experimental agents improve the PI3 K/Akt signaling pathway in PD models. However, preclinical studies'findings do not completely translate into clinical studies, and most experimental PI3k/Akt activators did not enter the clinical trials [79]. Therefore, repurposing approved drugs with stimulating properties on the PI3 K/AKT signaling pathway may attenuate the development and progression of PD (Table 2).

**Table 2:** Role of PI3k/Akt activators in PD

PI3k/Akt activators	Findings	Ref
Pioglitazone	• Pioglitazone prevents Aβ42 accumulation in the hippocampus by activating IDE and PI3 K/AKT signaling pathways. The administration of PPARγ antagonist G9662 attenuates the neuroprotective effect of pioglitazone in the AD mouse model	[80]
	• Pioglitazone attenuates the development of BIR and tauopathy in rats with experimental T2D and PD by activating the PI3 K/AKT signaling pathway	[81, 82]
	• In animal models, pioglitazone attenuates hippocampal neurodegeneration and cognitive impairment by activating the hippocampal PI3 K/AKT signaling pathway	[83]
Fenofibrate	• Fenofibrate prevents hippocampal insulin resistance through the induction of the expression of the PI3 K/AKT signaling pathway	[84]
Nicorandil	• Nicorandil improves cell survival by activating the expression of the PI3 K/AKT signaling pathway in SH-SY5Y cells. Applying PI3 K/AKT inhibitor LY294002 prevents the neuroprotective effect of nicorandil	[85]
Metformin	• Metformin attenuates the accumulation of Aβ and phosphorylated tau protein in the hippocampus. It improves cognitive function by activating AD models' PI3 K/AKT signaling pathway	[86, 87]
	• Metformin has a neuroprotective effect against the 6-OHDA PD model by activating the AMPK/BDNF signaling pathway	[88]
Statins	• Atorvastatin and simvastatin attenuate experimental intracerebral hemorrhage by improving growth factors and brain PI3 K/AKT signaling pathways	[89]
	• Atorvastatin enhances neurite outgrowth in primary cortical neurons by inducing the expression of the PI3 K/AKT signaling pathway	[90]
	• Simvastatin attenuates Aβ-induced hippocampal neurotoxicity by activating the transgenic AD model's PI3 K/AKT signaling pathway	[91]

### Pioglitazone

Pioglitazone is an antidiabetic drug used in the management of T2D. It acts by activating peroxisome proliferator activator-activated receptor gamma (PPARγ) and decreasing the expression of genes intricate in the control of blood glucose [92, 93]. Pioglitazone attenuates the development of peripheral insulin resistance and chronic hyperglycemia in T2D [92, 93]. It has been suggested that pioglitazone delays the onset of cognitive impairment and AD by reducing the development of BIR [94]. However, this suggestion was not confirmed by clinical trials [95]. Many preclinical studies highlighted the neuroprotective effects of pioglitazone against the development of PD neuropathology [96, 97]. PPARγ agonists attenuate neurodegeneration of the dopaminergic neurons in the SNpc by inhibiting microglia in the MPTP mouse model [96]. Pioglitazone also improves cognitive impairment by inhibiting oxidative stress and inflammation in the PD mouse model [97]. Analysis of preclinical studies highlighted that pioglitazone effectively treats PD models [98]. However, a phase II clinical trial showed that pioglitazone was ineffective in reducing symptoms in PD patients [99]. Cheng et al. [100] found that pioglitazone was more effective than other antidiabetic glipizide in reducing AD risk, according to the genetic analysis of a population-based study. A systematic review by Basutkar et al. [101] illustrated that pioglitazone reduced AD risk. Furthermore, a recent systematic review and meta-analysis indicated that prolonged use of pioglitazone reduced PD risk in T2D patients [102].

The neuroprotective effect of pioglitazone against AD neuropathology is related to diverse mechanisms. One of the important mechanisms is the activation of the PI3 K/AKT signaling pathway. A preclinical study found that pioglitazone prevents Aβ42 accumulation in the hippocampus by activating IDE and PI3 K/AKT signaling pathways. Supporting this finding, the administration of PPARγ antagonist G9662 attenuates the neuroprotective effect of pioglitazone in the AD mouse model [80]. Furthermore, pioglitazone attenuates the development of BIR and tauopathy in rats with experimental T2D and PD by activating the PI3 K/AKT signaling pathway. Hyperphosphorylation of tau protein in pioglitazone-treated rats with T2D was improved. Pioglitazone can ameliorate BIR and decrease tau-protein hyperphosphorylation, but cannot increase brain insulin levels [81, 82]. Thus, targeting metabolic dysfunction in PD with pioglitazone can improve motor and cognitive functions and produce significant neuroprotective effects in PD patients. In addition, pioglitazone attenuates hippocampal neurodegeneration and cognitive impairment by activating the hippocampal PI3 K/AKT signaling pathway and other signaling pathways, such as the mTOR pathway, in animal models [83]. Therefore, pioglitazone, via activation of the brain PI3 K/AKT signaling pathway, can reduce the development of PD.

### Fenofibrate

Fenofibrate is a PPARα agonist drug used in treating mixed dyslipidemia and hypertriglyceridemia [103–106]. Besides, fenofibrate has pleiotropic anti-inflammatory, antioxidant, and antithrombotic effects. Fenofibrate is effective in diverse neurodegenerative diseases such as multiple sclerosis [107–109]. Many preclinical studies highlighted that fenofibrate reduced AD neuropathology by increasing clearance of Aβ by proteolysis or reducing Aβ production [110, 111]. Fenofibrate inhibits the activity of BACE-1, thereby reducing the generation of neurotoxic Aβ1–42 via activation of the PI3 K/AKT signaling pathway in transgenic mice [111]. Matsuda et al. [112] illustrated that fenofibrate reduced AD neuropathology by decreasing oxidative stress via activating the PI3 K/AKT signaling pathway in animal models. Notably, fenofibrate attenuates the development of BIR in the T2D rat model by increasing the PI3 K/AKT signaling pathway activity in the hippocampus [113].

Similarly, fenofibrate prevents the development of hippocampal insulin resistance by mitigating oxidative stress and inflammation in astrocytes through induction of the expression of the PI3 K/AKT signaling pathway [84]. Thus, by activating the brain PI3 K/AKT signaling pathway, fenofibrate may decrease PD's development and progression. Furthermore, fenofibrate and other PPARα agonists reduce dopaminergic neurodegeneration in the SNpc in the PD rat model via modulation of the cellular signaling pathway and suppression of oxidative stress. Animal models of PD have shown that neuroinflammation is one of the most important mechanisms involved in dopaminergic cell death, suggesting that fenofibrate attenuates MPTP-induced neurodegeneration [114]. Similarly, fenofibrate impedes the accumulation of α-Syn in the SNpc with subsequent amelioration of cognitive impairment and behavioral dysfunction in the rotenone PD model. Moreover, fenofibrate lessened the depletion of striatal dopamine and protected against dopaminergic neuronal death in the SNpc, signifying that fenofibrate mitigates the pathogenesis of PD [115]. Ogawa et al. [116] consistently showed that fenofibrate, via activation of PPARα and SIRT1 signaling, inhibits microglial activation and the release of pro-inflammatory cytokines in the murine microglia cell line. In addition, a selective PPARα modulator, pemafibrate, like other PPARα ligands, potently suppressed NF-κB phosphorylation and cytokine expression in microglial cells. PPARα knockdown significantly amplified LPS-induced cytokine expression. Pemafibrate-induced suppression of IL-6 expression was reversed by PPARα knockdown. However, suppression by fenofibrate was not reversed by PPARα knockdown but by SIRT1 knockdown [115]. Therefore, pemafibrate and fenofibrate similarly suppress microglial activation through distinct PPARα and SIRT1-dependent pathways. Consequently, fenofibrate, through stimulation of the brain PI3 K/AKT signaling pathway, can decrease the development of PD.

### Nicorandil

Nicorandil is an ATP-sensitive potassium channel opener that protects against cardiac and brain ischemic injury [117]. Findings from an in vitro study observed that nicorandil improves cell survival by activating the expression of the PI3 K/AKT signaling pathway in SH-SY5Y cells expressing mutant APP. Applying PI3 K/AKT inhibitor LY294002 prevents the neuroprotective effect of nicorandil [85]. In addition, nicorandil improves cerebral blood flow and enhances synaptogenesis in ischemic rat models [118]. Additionally, preclinical studies found that nicorandil attenuates diabetic cardiomyopathy and cardiac ischemic perfusion injury by activating the PI3 K/AKT signaling pathway [119, 120]. Therefore, by activating the PI3 K/AKT signaling pathway, nicorandil may improve PD neuropathology and other neurodegenerative diseases. It has been specified that nicorandil, by tempering chronic cerebral hypoperfusion, averts the development of vascular dementia in mice [121]. Zhao, Gong [122] found that chronic cerebral hypoperfusion can cause AD-like pathology and neurodegeneration in experimental animals. Nicorandil also attenuates the development of peripheral insulin resistance by inhibiting oxidative stress and ER stress [123]. Therefore, by activating the PI3 K/AKT signaling pathway, nicorandil can reduce the pathogenesis of PD and other neurodegenerative diseases. An in vitro study demonstrated that nicorandil minimizes the severity of neurodegeneration by reducing oxidative stress in the SH-SY5Y cell line [124]. In addition, nicorandil enhances post-stroke neurological dysfunction by inhibiting the development of neuroinflammation via suppression of microglia and the expression of NF-κB [125]. Therefore, nicorandil can reduce PD severity by activating the PI3 K/AKT signaling pathway and suppressing neuroinflammation.

### Metformin

Metformin is a first-line treatment for T2D; it acts as an insulin-sensitizing drug and inhibits hepatic glucose output by activating AMPK [126, 127]. Metformin has anti-inflammatory, antioxidant, and antiapoptotic effects [128–130]. Furthermore, metformin may effectively manage AD by inhibiting the formation of amyloid plaque and NFTs [131]. Metformin has a neuroprotective effect against brain ischemic reperfusion injury by activating the PI3 K/AKT signaling pathway in an ischemic rat model [132–139]. Likewise, inhibition of the PI3 K/AKT signaling pathway precludes the neuroprotective effect of metformin against brain ischemic injury [140]. In addition, metformin attenuates the accumulation of Aβ and phosphorylated tau protein in the hippocampus. It improves cognitive function by activating AD models' PI3 K/AKT signaling pathway [86, 87].

Metformin activation of the PI3 K/AKT signaling pathway also reduced the hippocampus's Aβ and phosphorylated tau protein load and improved memory function in diabetic mice [141]. Likewise, metformin prevents the development and progression of BIR by attenuating mitochondrial dysfunction in mice with a high-fat diet [142]. In the bargain, metformin, in combination with exercise, prevents the development of BIR by reducing mitochondrial dysfunction in mice with high-fat diets [143]. It has been observed that metformin affects PD neuropathology at different levels; it can regulate oxidative stress and inflammation in the dopaminergic neurons of the SNpc [144]. Alrouji et al. [145] supposed that metformin has a double-edged effect on the pathogenesis of PD that may be beneficial or detrimental. Metformin controls astrocyte reactivity in PD and normal aging. Metformin has a neuroprotective effect against the 6-OHDA PD model by activating the AMPK/BDNF signaling pathway [88].

**Fig. 7:**
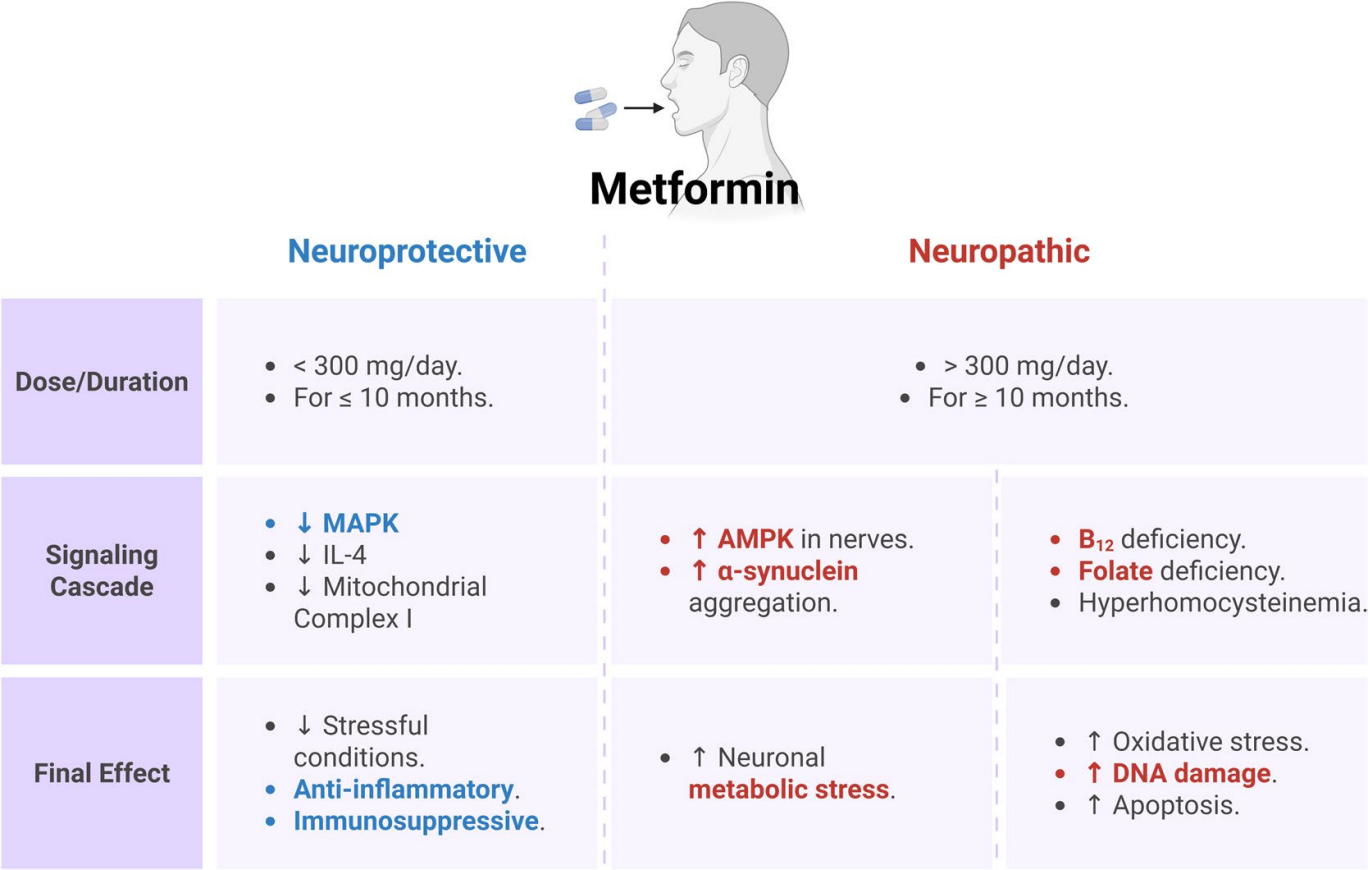
The double-edged effect of metformin in the pathogenesis of PD

Moreover, the neuroprotective effect of metformin against experimental PD is also mediated by activating autophagy in the SNpc [146]. A cohort and longitudinal study found that prolonged use of low-dose metformin reduced PD risk in T2D patients [147]. Metformin may effectively treat PD patients by activating the brain PI3 K/AKT signaling pathway (Fig. 7).

### Statins

Statins are cholesterol-lowering drugs extensively used to manage dyslipidemia and cardiovascular disorders [148]. Statins inhibit cholesterol biosynthesis by constraining liver hydroxyl methyl-gutaryl coenzyme A (HMG-CoA) reductase [149]. Statins have pleiotropic effects through cholesterol-dependent and cholesterol-independent mechanisms [150–154]. Moreover, statins have neuroprotective effects against neurodegenerative diseases [155–159]. Furthermore, atorvastatin and simvastatin attenuate experimental intracerebral hemorrhage by improving growth factors and brain PI3 K/AKT signaling pathways [89]. Jin et al. [90] found that atorvastatin enhances neurite outgrowth in primary cortical neurons by inducing the expression of the PI3 K/AKT signaling pathway. Besides, simvastatin attenuates Aβ-induced hippocampal neurotoxicity by activating the transgenic AD model's PI3 K/AKT signaling pathway [91]. Therefore, statins may improve PD neuropathology by activating the brain PI3 K/AKT signaling pathway. It has been proposed that statins can attenuate PD neuropathology by reducing oxidative stress, neuroinflammation, and modulation of the PI3 K/AKT signaling pathway [157]. A randomized clinical trial highlighted that simvastatin was futile in treating PD. In this randomized clinical trial, simvastatin was ineffective as a disease-modifying therapy in patients with PD of moderate severity, providing no evidence to support proceeding to phase 3 trials [160]. In addition, a longitudinal study found that statins may increase PD risk. A survival analysis showed that the rate of dementia conversion was significantly higher in PD patients with statins than in those without. Mediation analyses discovered that the effect of statin treatment on baseline dopamine transporter availability and longitudinal outcome was not mediated by total cholesterol levels [161]. Therefore, statin use may have a detrimental effect on baseline nigrostriatal dopamine degeneration and long-term outcomes in PD patients.

Furthermore, a longitudinal study illustrated that hydrophilic statins exaggerate the development of PD non-motor symptoms [162]. Conversely, another longitudinal study revealed that prolonged use of statins reduced PD motor symptoms [163]. Likewise, evidence from observational studies highlighted that statins improve PD symptoms and reduce PD risk [164]. These controversial findings might be due to the heterogeneity of the studies and bias in the selection of PD patients. Consequently, a systematic review of meta-analysis showed that statins reduce PD and improve PD symptoms [165]. These verdicts indicated that statins could improve PD neuropathology by modulating many signaling pathways, including PI3 K/AKT.

**Fig. 8:**
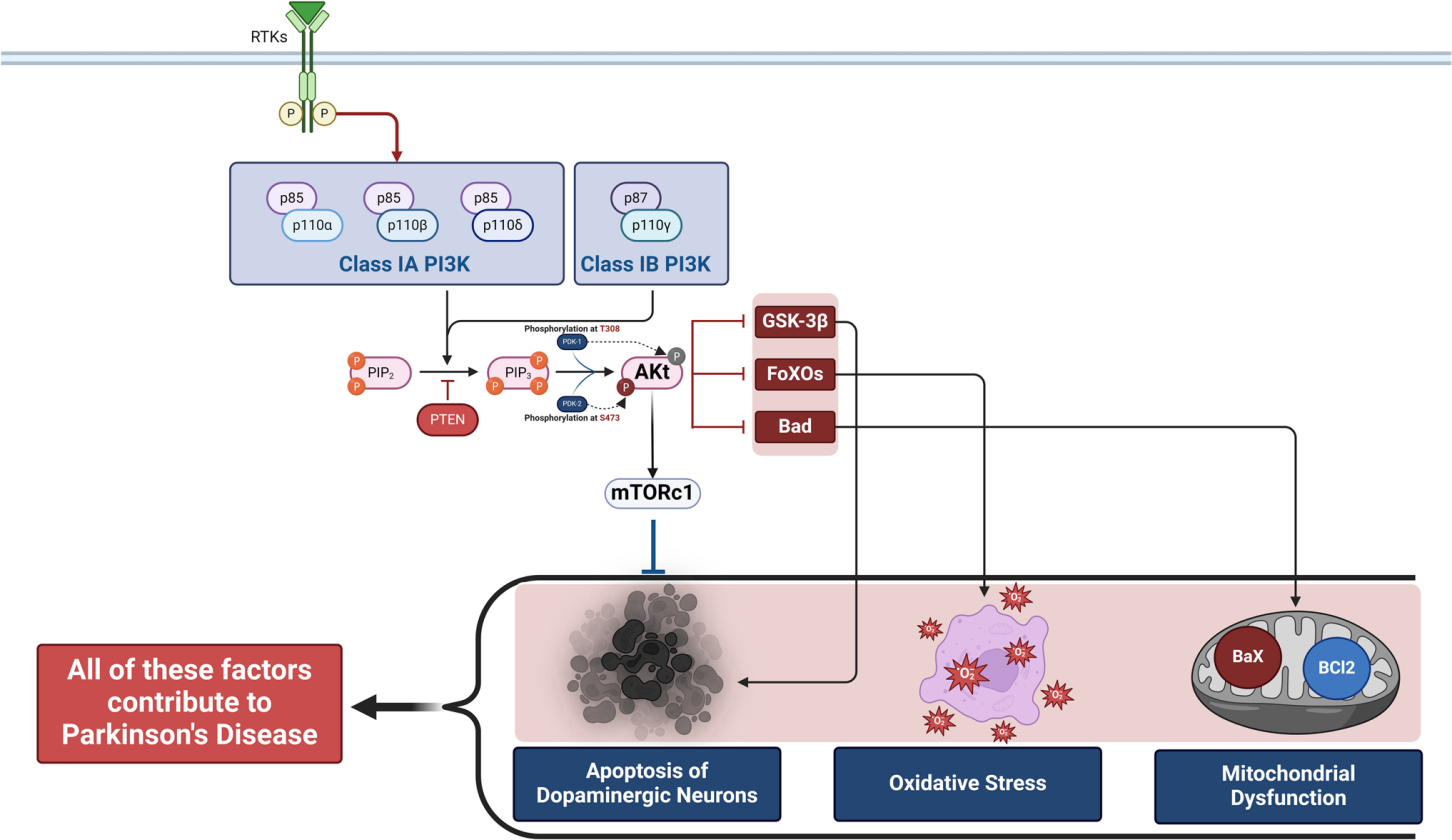
PI3 K/AKT activators in PD

Although promising, clinical studies with PI3 K/AKT activators in PD have shown inconsistent outcomes. Constraints include inadequate bioavailability, unintended off-target effects, and inconsistent effectiveness across diverse patient groups [166]. For example, pioglitazone and metformin may exhibit advantageous anti-inflammatory properties but have hazards of hypoglycemia and restricted blood–brain barrier (BBB) penetration [167]. Therefore, activators of PI3 K/AKT signaling pathways such as pioglitazone, fenofibrate, nicorandil, metformin, and statins could be effective in the management of PD need (Fig. 8) but need further studies.

## GSK3β Inhibitors in PD

It has been revealed that suppression of GSK3β signaling can temper the development and progression of PD by preventing neurodegeneration [168]. Nevertheless, GSK3β inhibitors are non-selective and linked with chronic neurotoxicity. The selectivity, potency, and safety of the GSK-3β inhibitors are vital for consideration as a potential therapeutic alternative. Several research studies have been carried out in search of novel options. Allosteric GSK-3β inhibitors are being pursued as more selective and safer drugs as alternative inhibitors. Allosteric inhibitors have a binding site that is different from that of the ATP pocket. GSK-3β Allosteric inhibitors have more advantages than other GSK-3β inhibitors and could open new avenues [169]. Likewise, most clinical trials revealed controversial and conflicting results from using GSK3β inhibitors [170]. Besides, a GSK3β inhibitor SB216763 can cause progressive neuronal apoptosis and neurotoxicity by broad-spectrum and non-specific inhibition of neuronal GSK3β [171]. Furthermore, GSK3β inhibitors have poor bioavailability, less cross the BBB, and increased risk of hypoglycemia and malignancy [172]. Thus, repurposing approved drugs that have suppressing properties on the GSK3β signaling pathway may lessen the development and progression of PD (Table 3).

**Table 3:** Role of GSK3β inhibitors in PD

GSK3β inhibitors	Findings	Ref
Lithium	• Lithium blocks GSK3β directly or indirectly by inhibiting the mTOR signaling pathway	[173]
	• Lithium attenuates PD neuropathology by inhibiting the expression of GSK3β, inducing neuronal autophagy, inhibition of neuroinflammation, oxidative stress, and the accumulation of α-Syn in the PD model	[174]
	• Lithium attenuates MPTP-induced dopaminergic neurodegeneration by inhibiting the aggregation of α-Syn and the GSK3β signaling pathway in the SNpc	[175]
Famotidine	• Famotidine has a neuroprotective effect by inhibiting exaggerated GSK3β activity in the SH-SY5Y cell line	[176]
Naproxen	• Naproxen reduces blood glucose by constraining the expression of GSK3β	[177]
Metformin	• Metformin attenuates Aβ accumulation in animal models by inhibiting the expression and the activity of GSK3β	[178]
Mevastatin	• Mevastatin prevents Aβ-induced neurotoxicity by suppressing GSK3β expression in SK-N-MC neuronal cells	[90, 178]

### Lithium

Lithium is the only GSK3β inhibitor used clinically as a mood-stabilizing agent [179]. However, prolonged use of lithium was not associated with the risk of hypoglycemia and malignancy because it inhibits only 25% of GSK3β and does not induce the Wnt/β-catenin signaling pathway, which increases the risk of cancer. Lithium blocks GSK3β directly or indirectly by inhibiting the mTOR signaling pathway [173]. Various studies emphasized that lithium reduced AD neuropathology by inhibiting the deposition of Aβ and tau protein in transgenic mice [180]. Furthermore, lithium may effectively manage AD by inhibiting oxidative stress and neuroinflammation and activating neuronal autophagy [181]. A systematic review and meta-analysis disclosed that lithium is effective against cognitive impairment and AD [182]. In addition, lithium attenuates PD neuropathology by inhibiting the expression of GSK3β in the SNpc. Lithium, by inducing neuronal autophagy, can reduce neuroinflammation, oxidative stress, and the accumulation of α-Syn in the PD model via inhibition of GSK3β [174]. Analysis and systematic review illustrated that lithium reduces neurodegeneration in PD models [183]. Zhao et al. [175] found that lithium attenuates MPTP-induced dopaminergic neurodegeneration by constraining the aggregation of α-Syn by inhibiting the GSK3β signaling pathway in the SNpc. Interestingly, higher concentrations of lithium in tobacco smoking account for a decreased incidence of PD in smoker subjects [184]. Nevertheless, lithium is not appropriate for long-term use due to the risk of toxicity and the development of adverse events [185].

### Famotidine

Famotidine is a histamine receptor 2 antagonist indicated in treating peptic ulcers and dyspepsia. Famotidine can cross the BBB and has a neuroprotective effect by inhibiting exaggerated GSK3β activity in the SH-SY5Y cell line [176]. In addition, famotidine regulates non-motor symptoms in PD patients and attenuates L-Dopa-induced dyskinesia [186, 187]. A placebo-controlled clinical trial observed that famotidine effectively mitigated cognitive impairment in COVID-19 patients compared to the placebo [188]. Currently, there are no clinical trials or clinical studies regarding the effect of famotidine on PD risk. A pilot study illustrated that histamine receptor 2 antagonist nizatidine can attenuate non-motor symptoms in patients with PD [189].

### Naproxen

Naproxen is a non-steroidal anti-inflammatory drug (NSAID) acting by non-selective inhibition of cyclooxygenase (COX) [190]. Findings from preclinical studies exemplified that naproxen actively reduced blood glucose by constraining the expression of GSK3β [177]. Furthermore, naproxen has an anticancer effect by suppressing GSK3β and Wnt/β-catenin signaling pathways [191]. Naproxen and other NSAIDs have been suggested to be effective in different neurodegenerative diseases such as AD and PD [192]. A previous randomized clinical trial showed that naproxen was ineffective in mitigating cognitive impairment and in reducing AD risk [193]. In addition, a randomized clinical trial showed that naproxen failed to produce a clinical benefit against AD risk [194]. A pilot study found that the NSAID diclofenac was more effective in reducing AD risk compared to naproxen, suggesting a class-dependent neuroprotective effect of NSAIDs against AD. Generally, NSAIDs attenuate AD neuropathology by inhibiting microglia, releasing pro-inflammatory cytokines, and developing neuroinflammation [195]. It has been stated that NSAIDs did not affect PD, according to the findings from a population-based study [196]. A retrospective cohort study indicated that prolonged use of NSAIDs was ineffective in reducing PD risk [197]. Accordingly, future studies are recommended to clarify the potential role of NSAIDs in the management of PD.

### Others Inhibitors

It has been stated that a small molecule inhibitor, tideglusib, irreversibly inhibits GSK3β activity and has a neuroprotective effect against PD and other neurodegenerative diseases in preclinical studies [78, 198, 199]. However, tideglusib was ineffective in mitigating cognitive impairment in AD patients [200]. A systematic review and meta-analysis showed that tideglusib and other GSK3β inhibitors were ineffective in managing cognitive impairment and AD [201]. On the other hand, metformin, through the AMPK pathway, inhibits the activity of GSK3β in the PD model [78]. Metformin attenuates Aβ accumulation in animal models by inhibiting the expression and the activity of GSK3β [178].

Furthermore, metformin reduced the severity of neurotoxicity in rat models by inhibiting the activity of GSK3β [202, 203]. Moreover, statins inhibit the activity of GSK3β and improve neurite outgrowth in primary cortical neurons through activation of PI3 K/AKT [203]. Jin et al. [90] that mevastatin prevents Aβ-induced neurotoxicity by suppressing GSK3β expression in SK-N-MC neuronal cells.

Finally, a natural product, curcumin, has been observed to attenuate Aβ-induced neurotoxicity by suppressing GSK3β expression and stimulating the PI3 K/AKT signaling pathway. Direct intracerebroventricular administration of curcumin-loaded nano-capsules reduced Aβ-induced cognitive impairment in rats by inhibiting GSK3β expression and improving synaptic plasticity [204]. Moreover, curcumin activates Wnt/β-catinine and restores synaptic plasticity by inhibiting GSK3β expression in the SHSY5Y cell line [204, 205]. Preclinical and clinical studies revealed that curcumin improves PD neuropathology [206, 207].

Taken together, PI3 K/AKT activators and GSK3β inhibitors play a role in mitigating cognitive impairment and PD neuropathology. Dual PI3 K/AKT activators and GSK3β inhibitors, such as statins and metformin, seem more appropriate in treating PD. Consequently, searching for a specific agent targeting PI3 K/AKT/GSK3β is suggested in this context.

## Discussion

PD is a complex neurological disease with ill-defined underlying neuropathology. The definition of PD is changing with the increase of clinical phenomenology and improved understanding of environmental and genetic effects that impact the pathogenesis of the disease at the cellular and molecular levels [208]. This has led to arguments and discussions, with, as yet, no general acceptance of the direction that change should take either at the level of diagnosis or of what should and should not be protected under the umbrella of PD [208]. Furthermore, anti-PD medications such as L-DOPA and other dopaminergic medications significantly improve the motor symptoms and quality of life of patients with PD in the early stages of the disease. However, once the honeymoon period has waned, usually after a few years of dopaminergic therapy, patients become progressively more disabled despite an ever more complex combination of available anti-PD medications. Sooner or later, PD patients suffer from L-DOPA resistance, characterized by motor and non-motor dysfunctions [209–212]. Therefore, the current anti-PD medications cannot be considered ideal concerning both efficacy and safety. Thus, targeting other signaling pathways involved in the pathogenesis of PD, such as PI3 K/AKT/GSK3β, is reasonable. In PD, PI3 K/AKT/GSK3β is dysregulated, characterized by overactivation of neuro-detrimental GSK3β and down-regulation of the neuroprotective PI3 K/AKT signaling with subsequent neurodegeneration. Therefore, applications of PI3 K/AKT activators, GSK3β inhibitors, and dual PI3 K/AKT activators and GSK3β inhibitors could effectively manage PD patients who do not respond to conventional anti-PD medications [213]. PI3 K/AKT activators such as pioglitazone, fenofibrate, nicorandil, metformin, and statins are FDA-approved drugs that can cross the BBB with promising efficacy and safety. However, these drugs are not specific for the brain PI3 K/AKT signaling pathway. Mounting studies designate that some natural products can play a neuroprotective role by activating the PI3 K/AKT pathway, providing a practical resource for discovering potential therapeutic drugs [29, 206, 207]. Notably, most natural medicines, such as curcumin, that target the brain PI3 K/AKT pathway have poor BBB penetration, limiting their neuroprotective effects in PD and other neurodegenerative diseases [214].

On the other hand, GSK3β inhibitors such as lithium, famotidine, and naproxen have limited efficacy and safety, mainly with lithium, which is associated with long-term severe adverse effects and toxicity [185]. In addition, small molecule inhibitors, such as tideglusib, inhibit GSK3β activity and have a neuroprotective effect against PD in preclinical studies [78, 198, 199] but not in clinical studies [200]. Interestingly, GSK3β and its signaling pathway hold great promise as a therapeutic target for many neurological disorders. Nevertheless, due to the wide range of GSK3β cellular targets, global kinase inhibition leads to severe side effects, and GSK3β inhibitors rarely reach Phase-2 clinical trials [215]. Therefore, selective modulation of a specific cellular pool of GSK3β or specific down- or upstream kinase partners might provide more efficient anti-PD therapy.

Dual PI3 K/AKT activators and GSK3β inhibitors, such as statins and metformin, seem more appropriate for treating PD. In addition, statins and metformin have direct neuroprotective effects regardless of the modulation of PI3 K/AKT/GSK3β [213]. Remarkably, statins and metformin reduce PD risk by attenuating the neurodetrimental effects of dyslipidemia and T2D, respectively [163, 216].

Importantly, validated biomarkers of the brain PI3 K/AKT/GSK3β signaling pathway in PD are essential to determine the therapeutic efficacy of PI3 K/AKT activators and/or GSK3β inhibitors. Transmission electron microscopy and scanning electron microscopy are used to evaluate the structures of the cerebral microvasculature [213]. They thus can be used to estimate the expression of the brain PI3 K/AKT/GSK3β signaling pathway. Furthermore, peripheral biomarkers of the PI3 K/AKT/GSK3β signaling pathway, such as serum levels and the expression of PI3 K/AKT and GSK3β in the peripheral blood, may reflect brain expression of PI3 K/AKT/GSK3β signaling pathway in PD.

The present review had many limitations, such as targeting the common upstream and downstream of the PI3 K/AKT/GSK3β signaling pathway, which was not fully discussed. In addition, findings of the present review regarding the role and targeting of the PI3 K/AKT/GSK3β signaling pathway were according with the preclinical studies that are not completely translated into the clinical settings. Therefore, preclinical studies and clinical trials regarding the efficacy and safety of the selective PI3 K/AKT activators and GSK3β inhibitors in PD are recommended.

## Future Directions

Developing selective modulators for the PI3 K/AKT and GSK3β pathways should be the primary focus of future research. Recent research indicates that dual-function agents, particularly those that inhibit GSK3β and activate PI3 K/AKT, may provide more effective therapeutic effects with fewer adverse effects [217]. Moreover, the limitations of current therapies, such as bioavailability issues, BBB penetration, and long-term safety, are warranted in this regard. More importantly, the PI3 K/Akt/GSK3β signaling cascade is worth additional examination in in vitro and in vivo trials, providing a new strategy for intervention in PD patients. Excitingly, future research directions expand on the development of targeted drugs, biomarkers based on patient stratification, the long-term safety and efficacy of dual-pathway modulators (PI3 K/AKT activators and GSK3β inhibitors) in PD, and the possibility of individualized treatment are warranted in this regard.

## Conclusions and Future Perspectives

In PD, the PI3 K/AKT/GSK3β signaling pathway is dysregulated and contributes to the development of cognitive impairment by exacerbating Aβ and tau protein phosphorylation. In particular, the PI3 K/AKT signaling pathway is deregulated while the GSK3β signaling pathway is upregulated, leading to progressive neurodegeneration of the dopaminergic neurons in SNpc. Therefore, activation of the PI3 K/AKT signaling pathway and suppression of GSK3β may be beneficial in managing PD. Activators of PI3 K/AKT signaling pathways, such as pioglitazone, fenofibrate, nicorandil, metformin, and statins, may effectively mitigate the pathogenesis of PD. Besides, GSK3β inhibitors can reduce PD neuropathology by inhibiting oxidative stress and neuroinflammation.

Taken together, PI3 K/AKT activators and GSK3β inhibitors play a role in alleviating cognitive impairment and PD severity. Dual PI3 K/AKT activators and GSK3β inhibitors, such as statins and metformin, seem more appropriate in treating PD. Consequently, searching for specific agents in future clinical trials and studies targeting PI3 K/AKT/GSK3β is suggested. Furthermore, preclinical models such as genetic and toxin-induced PD are needed to explore the most affected arm of PD's PI3 K/AKT/GSK3β signaling pathway. Additionally, modern translational neuroscience goals, precision medicine, and dual-target therapies regarding sex-dependent effects are recommended in this context.

## Data Availability

The data that support the findings of this study are included within the manuscript or the supplementary data.
